# Comparison of clinical efficacy, immune response and postoperative adverse reactions of transurethral holmium laser lithotripsy and percutaneous nephrolithotomy in elderly patients with complex upper urinary tract renal calculi: a retrospective cohort study

**DOI:** 10.3389/fsurg.2025.1588563

**Published:** 2025-10-29

**Authors:** Dian Fu, Rui Chen, Xiaoming Yi, Ding Wu, Haowei He, Ping Li, Wenquan Zhou, Jingping Ge, Wen Cheng

**Affiliations:** 1 Department of Urology, Jingling College of Clinical Medicine, Nanjing Medical University, Nanjing, China; 2 Department of Urology, The Ninth Medical Center, Chinese PLA General Hospital, Beijing, China

**Keywords:** transurethral holmium laser lithotripsy, retrograde intrarenal surgery (RIRS), PCNL, elderly patients with complex upper urinary tract renal calculi, immune reaction, postoperative adverse reactions

## Abstract

**Objectives:**

This study aimed to evaluate the clinical efficacy, immune response, and postoperative adverse effects of transurethral holmium laser lithotripsy (RIRS) vs. percutaneous nephrolithotomy (PCNL) in elderly patients with complex upper urinary tract renal calculi.

**Methods:**

A retrospective analysis was conducted on 70 elderly patients treated from January 2020 to January 2021. The control group (*n* = 32) underwent PCNL, while the observation group (*n* = 38) received transurethral holmium laser lithotripsy. Pre- and post-operative comparisons included serum creatinine (Scr), cystatin-C, kidney injury molecule-1 (KIM-1), immune indices, thyroxine (TH), and urokinase (UK). Stone clearance rates and adverse reactions were also assessed.

**Results:**

The observation group showed less bleeding, shorter hospital stays, higher hemoglobin decrease, and longer operation time (*P* < 0.05). Higher stone clearance rates were observed in the RIRS group at 86.84% and 76.32% for first and second stages, compared to 65.64% and 53.13% in the PCNL group. Postoperatively, Scr, Cys-C, and KIM-1 levels were lower in the RIRS group. Both groups exhibited decreased CD4+ and CD4+/CD8+, increased CD8+, reduced TH, and elevated UK levels post-surgery (*P* < 0.05). Adverse reactions were similar between groups.

**Conclusions:**

For elderly patients with complex renal calculi, transurethral holmium laser lithotripsy offers superior stone clearance and reduced renal damage compared to PCNL, despite a longer operation time. Consideration of individual patient conditions is crucial for selecting the optimal procedure.

## 1 Introduction

Urinary calculi represent a common urological condition, affecting 5%–10% of the global population, with kidney stones making up 40%–50% of these cases (1). In China, over 80% of urinary stone cases are kidney stones (2–4), and incidence rates have been increasing annually by 1%–5% (5–7). Factors including gender, body mass index (BMI), ethnicity, geographical environment, and genetic susceptibility significantly impact stone formation, primarily due to urine supersaturation with crystal salts (8). Complex upper urinary tract stones involve diverse microbial communities, such as Lactobacillus, Enterobacteriaceae, and Bifidobacterium, influencing stone development (9, 10). Accurate diagnosis through methods like urinary color ultrasound, urological plain film, urinary CT, and reconstruction is essential for tailoring effective treatment plans (11).

Traditional open surgery for complex stones has largely been replaced by minimally invasive techniques, such as extracorporeal shock wave lithotripsy (ESWL), percutaneous nephrolithotomy (PCNL) (12–14), and retrograde intrarenal surgery (RIRS) (15). PCNL is recommended by the European Association of Urology (EAU), American Urological Association (AUA), and Chinese Urological Association (CUA) guidelines for treating complex cases despite its complications, including bleeding and organ damage (16–18). RIRS offers a less invasive alternative with shorter hospital stays and recovery times (19–21).

In elderly patients with complex upper urinary tract renal calculi, there is no consensus on whether PCNL or holmium laser lithotripsy via soft ureteroscopy is more effective and safer (22, 23). This study aims to provide theoretical guidance by comparing these treatments in terms of clinical outcomes, immune responses, and postoperative adverse effects. Further research is necessary to determine the optimal treatment approach for these patients, considering the complexity and potential risks involved (24–26).

## 2 Patients and methods

### 2.1 General information

From January 2020 to January 2021, a retrospective analysis was conducted on 70 elderly patients with complex upper urinary tract renal calculi treated at the hospital. The control group included 32 patients who received percutaneous nephrolithotomy (PCNL), while the observation group comprised 38 patients who underwent transurethral holmium laser lithotripsy. In the control group, there were 23 males and 9 females aged from 60 to 81 years old, with an average age of 66.83 ± 6.31 years. Their BMI ranged from 17.78 to 27.89 kg/m^2^, with a mean value of 24.07 ± 1.56 kg/m^2^. The maximum stone diameter was between 2.0 and 2.8 cm, averaging 2.51 ± 0.23 cm. Among these patients, 20 had stones on the left side and 12 on the right. Stone types included 14 cases of staghorn calculi, 11 cases of complete cast stones, and 7 cases of incomplete cast stones. Years of education ranged from 6 to 16 years, averaging 10.25 ± 1.27 years. In the observation group, there were 28 males and 10 females aged from 61 to 82 years old, with an average age of 66.43 ± 6.32 years. Their BMI ranged from 17.71 to 27.96 kg/m^2^, averaging 24.11 ± 1.59 kg/m^2^. Maximum stone diameters were between 2.1 and 3.0 cm, averaging 2.51 ± 0.23 cm. Twenty-one patients had stones on the left side and 17 on the right. Stone types included 13 cases of staghorn calculi, 17 cases of complete cast stones, and 8 cases of incomplete cast stones. Years of education ranged from 5 to 16 years, averaging 10.33 ± 1.29 years. Regarding gender composition, age, BMI index, stone diameter, stone location, stone type, years of education, and other general characteristics, no notable differences were observed (*P* > 0.05), indicating comparability between groups.

Inclusion criteria for the study required that the location, size, number, and nature of stones be clearly diagnosed by urinary ultrasound, KUB, IVP, and urinary system CT. Patients needed to be aged 60 years or older with complete medical records. All participants had single kidney stones meeting the indications for flexible ureteroscopy and PCNL surgery. Preoperative tests including blood routine, electrolytes, liver and kidney function, coagulation function, and cardiopulmonary function were required to show no abnormalities. Conditions such as hypertension and diabetes were required to be stable, allowing patients to tolerate the operation. Participants also needed to have clear consciousness and provide informed consent for the study.

Exclusion criteria encompassed patients with renal malignant tumors, ectopic kidneys, spongy kidneys, polycystic kidneys, pregnant kidneys, transplanted kidney stones, urethral strictures, and lower ureteral strictures, along with those having a history of multiple surgeries for renal calculi. Patients with uncontrolled severe urinary tract infections, severe liver dysfunction, blood system diseases, or heart, brain, and lung diseases unable to tolerate surgery were excluded. Additionally, patients with incomplete examination or medical records, cognitive impairments, mental system diseases, or those participating in other clinical studies were not included.

All patients underwent standardized preoperative assessment, including coagulation function tests (INR, aPTT, platelet count) and cardiovascular risk evaluation. While specific antithrombotic medication regimens (e.g., warfarin, DOACs, aspirin) were not retrospectively extracted, no patient exhibited coagulopathy (defined as INR >1.5 or platelets <100 × 10⁹ /L) or required blood product transfusion intraoperatively. Per institutional protocol, patients on chronic antithrombotics were managed via multidisciplinary consultation to balance thrombotic and bleeding risks, consistent with 2025 EAU Guidelines (Section 3.4.6).

### 2.2 Stone density assessment

While preoperative non-contrast CT scans were routinely performed for stone diagnosis and surgical planning, Hounsfield Unit (HU) measurements of stone density were not systematically recorded in the retrospective dataset. This represents a limitation in characterizing stone hardness, which is a known predictor of lithotripsy efficacy. However, all included cases exhibited radiologically confirmed complex stones (staghorn or complete/incomplete cast stones >2 cm) requiring advanced surgical management. The distribution of stone types (staghorn, complete/incomplete cast stones) showed no significant intergroup difference (*P* > 0.05*P* > 0.05, suggesting comparable baseline stone complexity between cohorts.

### 2.3 Treatment methods

All PCNL procedures utilized single-tract 16F access under fluoroscopic guidance. While multi-tract approaches or Endoscopic Combined IntraRenal Surgery (ECIRS) are increasingly adopted for complex calculi, this study reflects institutional protocols during 2020–2021 where single-tract mini-PCNL was standard for elderly patients with comorbidities. Stone fragmentation employed pneumatic/ballistic devices rather than ultrasonic or combination lithotripters. Notably, ECIRS—which integrates antegrade and retrograde access—was not routinely available at our center during the study period but is recognized for superior stone-free rates (SFR) in contemporary practice [cite EAU Guidelines 2025].

In the observation group, patients underwent PCNL with combined spinal-epidural anesthesia. The target renal calyx was punctured under x-ray guidance, and a 16F channel was established for stone extraction, using a ureteroscope. Lithotripsy was performed with Di laser or air pressure, and stones were removed using a stone basket. Postoperatively, both a nephrostomy tube and a double J tube were placed. The nephrostomy tube was removed one week post-operation, while the double J tube was removed 2–4 weeks later.

According to the 2025 EAU Guidelines on Urolithiasis, while PCNL remains the first-line treatment for stones >2 cm, flexible ureteroscopy may serve as an alternative in patients where anticoagulation cannot be safely interrupted, such as elderly individuals with cardiovascular comorbidities [EAU Guidelines 2025, Section 3.4.6]. This consideration may partially explain the favorable outcomes observed in our fURS group despite the complexity of the stones.

The control group received soft ureteroscopy with holmium laser lithotripsy under general anesthesia. Patients were placed in the bladder lithotomy position, and an endoscope was inserted under direct vision after placing a guide wire and ureteral sheath. A soft ureteroscope was then used to locate the stones, which were fragmented with a 200 μm Di laser fiber to sizes ≤3 mm and extracted using a stone basket. A 5F double J tube was placed at the end of the procedure and removed 2–4 weeks post-operation following confirmation of complete stone clearance via urinary tract plain film.

Postoperative x-ray examinations were conducted for both groups, and any residual stones were addressed with secondary surgical treatments as needed.

### 2.4 Observation index

Operation time, intraoperative blood loss, hospital stay, and decrease in hemoglobin (Hb) were calculated. Blood loss was determined using the weighing method: blood loss (g) = weight of gauze after absorption of blood (g)—dry gauze weight (g), with 1 g equivalent to 1 ml. Stone clearance rates were assessed via B-ultrasound and abdominal plain film five days post-operation. Successful stone clearance was defined as stone debris ≤4 mm, complete resolution of symptoms such as nausea, vomiting, and abdominal colic, and normalization of physical signs. The stone clearance rate was calculated as the number of successful clearances divided by the total number of cases multiplied by 100%.

Renal injury indices including serum creatinine (Scr), serum cystatin-C, and kidney injury molecule-1 (KIM-1) were measured from venous blood samples taken before surgery and two weeks post-operation. Immune indices CD4+ and CD8+ were detected using flow cytometry, with the CD4+/CD8+ ratio calculated. Thyroxine (TH) levels were measured by electrochemiluminescence (ECL), and urokinase (UK) levels by enzyme-linked immunosorbent assay (ELISA). All kits and instruments were sourced from Nanjing Sembega Biotechnology Co., Ltd., and Beckman Coulter, respectively. Assessments were conducted preoperatively and two weeks post-operation.

Renal injury was assessed via serum biomarkers (Scr, Cys-C, KIM-1) measured preoperatively and at 2 weeks postoperatively. These biomarkers were selected based on their validated roles:

#### 2.4.1 KIM-1

Sensitive early marker of tubular injury (elevated within 24–72 h post-insult).

#### 2.4.2 Scr/Cys-C

Reflect glomerular filtration rate (GFR) changes, with Cys-C being less influenced by muscle mass than Scr.

While this design captures acute surgical-induced kidney stress, it does not evaluate long-term functional outcomes. Post-discharge follow-up (e.g., 3–6 months) would be needed to assess chronic damage or recovery.

The incidence of adverse reactions, including fever, ureteral perforation, urinary tract infection, and bleeding within two weeks post-operation, was compared between groups, and the total incidence of adverse reactions was calculated. This comprehensive evaluation aimed to provide insights into the efficacy and safety of each treatment modality for complex upper urinary tract renal calculi.

### 2.5 Statistical analysis

The data were analyzed using SPSS 19.0 software. Measurement data with normal distribution and homogeneous variances are expressed as mean ± standard deviation (±s) (27).

For intra-group comparisons, a paired *t*-test was used.

For inter-group comparisons, an independent samples t-test was applied.

Enumeration data are presented as number and percentage (*n*, %), and were analyzed using the chi-square (*χ*^2^) test.

A *p*-value < 0.05 was considered statistically significant (28, 29).

## 3 Results

### 3.1 Comparison of clinical indexes

Compared with the control group, the observation group had less blood loss, shorter hospital stays, higher hemoglobin value, and longer operation time (*P* < 0.05). You can see all results in Table 1.

**Table 1:** The clinical indexes between the two groups $\left(\bar{x}\pm s\right)$.

Group	*N*	The length of operation(min)	Intraoperative bleeding volume	Hospitalization time	Hb decreasing value (g/L)
C group	32	82.14 ± 2.63	77.21 ± 2.83	5.93 ± 0.71	0.46 ± 0.21
O group	38	113.24 ± 3.71	25.83 ± 3.41	3.46 ± 0.32	13.27 ± 3.09
*t*		39.733	67.793	19.266	23.379
*P*		<0.05	<0.05	<0.05	<0.05

A total of 4 doctors participated in the surgeries in this study. PCNL was performed by doctors A and B (50 cases per year on average), and RIRS was performed by doctors C and D (80 cases per year on average). All doctors passed standardised assessments.

It is worth noting that the mean operative time in Group O (flexible ureteroscopy group) was 113.24 ± 3.71 min, which exceeds the 90 min recommendation from the 2025 EAU Guidelines on Urolithiasis (Section 3.4.6). This guideline emphasizes that prolonged operative times during ureteroscopy are associated with an increased risk of complications such as postoperative fever, sepsis, and ureteral injury. While our study demonstrated advantages in terms of reduced intraoperative bleeding and shorter hospitalization in Group O, the extended operative duration raises concerns regarding patient safety and should be carefully considered in clinical practice. Efforts should be made to optimize surgical efficiency—such as through enhanced preoperative planning, improved access techniques, or use of adjunctive technologies—to align with current best-practice standards and reduce potential morbidity.

### 3.2 The stone clearance rate in the first and second stage

**Figure 1:**
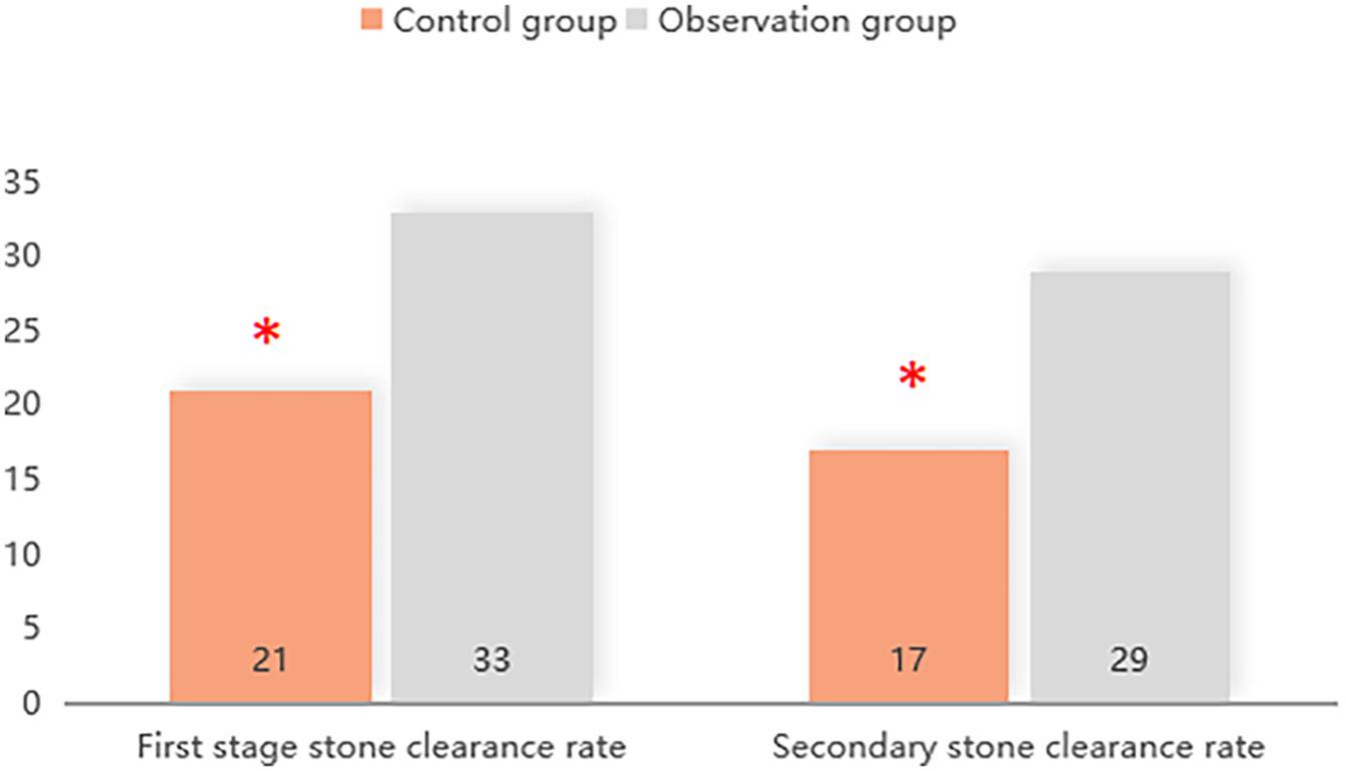
The first- and second-stage stone clearance rates in the observation group.

The first- and second-stage stone clearance rates in the observation group were 86.84% (33/38) and 76.32% (29/38) respectively, which were higher than 65.64% (21/32) and 53.13% (17/32) in the control group (*P* < 0.05). You can see all results in Figure 1.

### 3.3 Comparison of renal injury indexes

There exhibited no remarkable difference in the preoperative levels of Scr, Cys-C and KIM-1 (*P* > 0.05). After operation, the levels of Scr, Cys-C and KIM-1 in the observation group were lower (*P* < 0.05). You can see all results in Table 2.

**Table 2:** Renal function biomarkers before and after treatment.

Group	*N*	Scr (μmol/L)		Cys-C (μmol/L)		KIM-1 (pg/mL)	
		Before operation	After operation	Before operation	After operation	Before operation	After operation
C group	32	87.32 ± 12.71	80.72 ± 11.42^a^	1.17 ± 0.32	0.99 ± 0.13^a^	3.24 ± 0.61	0.87 ± 0.29^a^
O group	38	84.76 ± 12.53	74.25 ± 10.17^b^	1.23 ± 0.25	0.77 ± 0.12^b^	3.36 ± 0.73	0.53 ± 0.21^b^
*t*		0.046	2.507	0.880	7.356	0.738	5.676
*P*		>0.05	<0.05	>0.05	<0.05	>0.05	>0.05

^a^Comparison within the group before and after treatment, *P* < 0.05.

^b^Comparison between the two groups after treatment, *P* < 0.05.

### 3.4 The immune index levels before and after treatment

There exhibited no remarkable difference in the preoperative CD4^+^, CD8^+^, CD4^+^/CD8^+^ levels (*P* > 0.05). After surgery, the levels of CD4+ and CD4^+^/CD8^+^ were remarkably lower compared to before treatment (*P* < 0.05), while the level of CD8^+^ was remarkably higher compared to before surgery (*P* < 0.05). Additionally, the levels of CD4^+^ and CD4^+^/CD8^+^ in the control group were remarkably lower (*P* < 0.05). The level of CD8^+^ was remarkably higher (*P* < 0.05). You can see all the results in Table 3.

**Table 3:** Cd4+ and CD4+/CD8+ levels before and after treatment.

Group	*N*	CD4^+^ (%)		CD8^+^ (%)		CD4^+^/CD8^+^ (%)	
		Before operation	After operation	Before operation	After operation	Before operation	After operation
C group	32	41.98 ± 6.31	27.31 ± 3.58^a^	31.45 ± 3.51	38.71 ± 4.25^a^	1.35 ± 0.42	0.71 ± 0.35^a^
O group	38	42.35 ± 5.88	33.54 ± 4.03^b^	31.81 ± 3.46	33.56 ± 4.03^b^	1.32 ± 0.33	0.97 ± 0.22^b^
*t*		0.254	6.777	0.431	5.195	0.335	3.780
*P*		>0.05	<0.05	>0.05	<0.05	>0.05	>0.05

^a^Comparison within the group before and after treatment, *P* < 0.05.

^b^Comparison between the two groups after treatment, *P* < 0.05.

### 3.5 TH and UK levels before and after operation

There exhibited no remarkable difference in the preoperative TH and UK levels (*P* > 0.05). After surgery, compared to before treatment, the TH levels were remarkably lower (*P* < 0.05), while the UK levels were remarkably higher (*P* < 0.05). And the level of TH in the observation group was remarkably lower (*P* < 0.05), and the level of UK was remarkably higher (*P* < 0.05). You can see all the results in Table 4.

**Table 4:** TH and UK levels before and after treatment $\left[\bar{x}\pm s\right]$.

Group	*N*	TH (nmol/L)		UK (μg/L)	
		Before operation	After operation	Before operation	After operation
C group	32	238.81 ± 50.37	186.45 ± 35.73^a^	0.45 ± 0.12	0.71 ± 0.15^a^
O group	38	237.42 ± 52.83	154.73 ± 23.01^b^	0.41 ± 0.11	0.93 ± 0.13^b^
*t*		0.112	4.482	1.454	6.574
*P*		>0.05	<0.05	>0.05	<0.05

^a^Comparison within the group before and after treatment, *P* < 0.05.

^b^Comparison between the two groups after treatment, *P* < 0.05.

### 3.6 The postoperative adverse reactions

**Figure 2:**
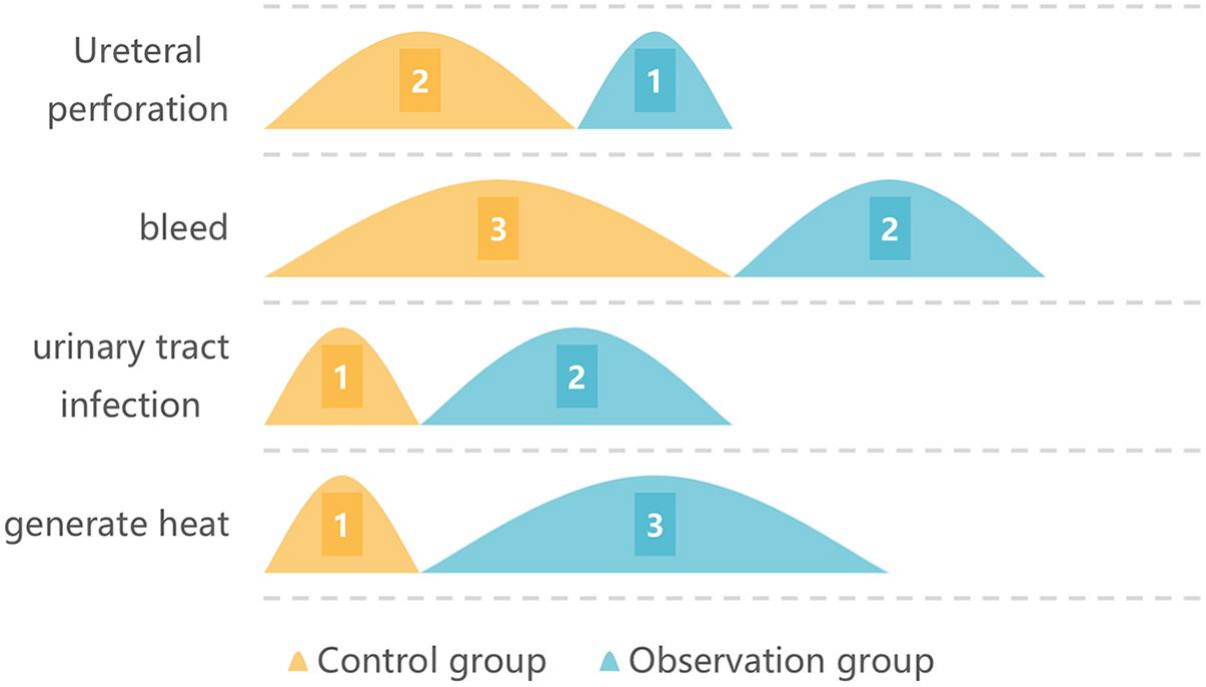
The incidence of postoperative adverse reactions.

In the control group, there were 1 people of fever, 1 people of urinary tract infection, 3 people of hemorrhage and 1 people of ureteral perforation. The incidence of postoperative adverse reactions was 21.87%. In the study group, there were 3 people of fever, 2 people of urinary tract infection, 2 people of hemorrhage and 1 people of ureteral perforation. The incidence of postoperative adverse reactions was 21.05%. No remarkable difference was observed in the incidence of postoperative adverse reactions (*P* > 0.05). You can see all the results in Figure 2 and Table 5.

**Table 5:** Clavien-Dindo classification of complications.

Group	Total complications	Grade I	Grade II	Grade IIIa	Grade IIIb	Grade IV/V
PCNL (*n* = 32)	6 (18.8%)	3	2	1^a^	0	0
RIRS (*n* = 38)	8 (21.1%)	5	2	1^b^	0	0

Hemorrhage managed conservatively (II), UTI treated with antibiotics (II), Fever resolving spontaneously (I).

a PCNL: 1 ureteral perforation requiring stent (IIIa).

b RIRS: 1 ureteral perforation requiring stent (IIIa).

## 4 Discussion

The comparative analysis of transurethral holmium laser lithotripsy (RIRS) and percutaneous nephrolithotomy (PCNL) in elderly patients with complex upper urinary tract calculi revealed several key findings. RIRS demonstrated superior stone-free rates (86.84% vs. 65.64%) and reduced renal injury, as evidenced by significantly lower postoperative levels of serum creatinine, cystatin-C, and kidney injury molecule-1. These advantages were accompanied by shorter hospital stays and less intraoperative blood loss, making RIRS particularly suitable for elderly patients with limited physiological reserve.

The immunological assessment showed significant postoperative changes in immune parameters, with CD4+ and CD4+/CD8+ ratios decreasing while CD8+ levels increased. These alterations were less pronounced in the RIRS group, suggesting this approach may cause less immunosuppressive stress. Biochemical markers including urokinase and thyroxine levels showed favorable changes following RIRS treatment, further supporting its clinical benefits.

Technical considerations significantly influenced outcomes. The single-tract 16F PCNL approach used in this study inherently limited visualization and fragment retrieval, while the absence of ultrasonic lithotripsy reduced dusting efficiency. In contrast, RIRS benefits from ureteral access sheaths and high-frequency holmium laser settings (0.8–1.2 J, 10–15 Hz) that facilitate efficient stone management. However, RIRS procedures required longer operative times (averaging 113.24 min), which carries known risks of complications.

While Endoscopic Combined Intrarenal Surgery (ECIRS) has emerged as the gold standard for complex stones with reported stone-free rates of 85%–95%, its application in elderly patients requires careful consideration. The technical demands, combined anesthesia requirements, and prolonged operative times may outweigh benefits for frail patients. In such cases, RIRS presents a favorable risk-benefit alternative.

Several limitations should be acknowledged. The study's focus on acute renal injury biomarkers (measured at 2 weeks postoperatively) without long-term follow-up prevents assessment of chronic kidney damage. The single-center design and relatively small sample size may affect generalizability. Future studies should incorporate extended follow-up periods, additional inflammatory markers such as IL-6 and CRP, and direct comparisons with ECIRS in elderly populations.

These findings support RIRS as an effective minimally invasive option for elderly patients with complex upper tract stones, particularly when ECIRS is contraindicated or unavailable. The choice between surgical approaches should be individualized, considering stone characteristics, patient comorbidities, and available surgical expertise. Further research is needed to establish long-term renal outcomes and refine patient selection criteria.

## 5 Conclusion

This retrospective cohort study demonstrates that transurethral holmium laser lithotripsy (RIRS) provides distinct advantages compared to single-tract mini-PCNL for elderly patients with complex upper urinary tract calculi. The RIRS group achieved significantly higher stone-free rates of 86.84% vs. 65.64% in the PCNL group during initial treatment. This superior efficacy stems from the precise dusting capability of holmium laser technology and the ability to minimize residual fragments through repeated access.

Postoperative renal function assessments revealed significantly lower levels of serum creatinine, cystatin-C, and kidney injury molecule-1 in the RIRS cohort. These biomarker profiles indicate reduced acute tubular and glomerular stress following retrograde intrarenal surgery. The clinical benefits extended to shorter hospitalization durations and decreased intraoperative blood loss, both critical considerations for elderly patients with limited physiological reserve.

The study identified longer operative times as the primary drawback of RIRS, averaging 113.24 min compared to PCNL. Prolonged endoscopic procedures carry established risks including ureteral injury and postoperative infection. While Endoscopic Combined Intrarenal Surgery (ECIRS) demonstrates superior stone clearance rates of 85%–95% in contemporary practice, its technical complexity and anesthesia requirements may present prohibitive risks for frail elderly patients.

Clinical decision-making should incorporate multiple factors including stone burden, anatomical considerations, and patient comorbidities. For high-risk elderly patients, RIRS represents an effective balance between stone clearance and procedural safety. In cases of extensive staghorn calculi, multi-tract PCNL or ECIRS may be preferable when supported by institutional expertise and patient fitness. Future investigations should incorporate longitudinal renal function monitoring and direct comparisons between RIRS and ECIRS in elderly populations.

The findings support RIRS as a viable minimally invasive alternative to single-tract PCNL for complex upper tract stones in elderly patients. Optimal treatment selection requires careful consideration of individual patient characteristics, stone complexity, and available surgical resources. Further research is needed to establish long-term renal outcomes and refine patient selection criteria for various surgical approaches.

## Data Availability

The original contributions presented in the study are included in the article/Supplementary Material, further inquiries can be directed to the corresponding authors.
